# How Is Stress Reduced by a Workplace Mindfulness Intervention? A Qualitative Study Conceptualising Experiences of Change

**DOI:** 10.1007/s12671-017-0790-2

**Published:** 2017-09-02

**Authors:** Siobhan Hugh-Jones, Sally Rose, Gina Z. Koutsopoulou, Ruth Simms-Ellis

**Affiliations:** 1 School of Psychology, University of Leeds, Leeds, LS2 9JT UK; 2 Staff Counselling and Psychological Support Service, University of Leeds, Leeds, LS2 9JT UK

**Keywords:** Mindfulness, Workplace interventions, Stress, Mechanisms of change, Qualitative

## Abstract

Mindfulness-based interventions are effective as curative and preventative approaches to psychological health. However, the mechanisms by which outcomes are secured from such interventions when delivered in the workplace, and to a stressed workforce, are not well understood. The aim of the present study was to elicit and analyse accounts from past participants of a workplace mindfulness intervention in order to generate a preliminary model of how positive benefits appear to be secured. In-depth, semi-structured interviews were completed with 21 employees of a higher education institution who had completed an eight-week intervention based on Mindfulness-Based Stress Reduction, adapted for the workplace. Interviews invited participants to recount their experiences of the intervention and its impact, if any, on their work life. Aspects of the interview data that pertained to intervention experience and positive benefits were analysed using a version of grounded theory, leading to the generation of a provisional model of how positive change occurred. The model suggests that discrete, temporal experiences build on each other to generate multiple, positive benefits. As anticipated in mindfulness-based interventions, enhanced attentional capacity was important, but our provisional model also suggests that resonance, self-care, detection of stress markers, perceiving choice, recovering self-agency and upward spiralling may be central mechanisms that lead to positive outcomes. Understanding mechanisms of change may help support participant engagement and trust in work-based mindfulness programmes, and enhance participants’ ability to apply mindfulness in their work life.

## Introduction

The inflammatory response triggered by persistent psychological stress has been implicated in virtually all chronic physical conditions (Cohen et al. 2007; Yusuf et al. 2004), and many mental health conditions (Garcia-Bueno et al. 2008). Enduring work-related stress is a major contributor to overall stress and meta-analyses of prospective studies indicate it is associated with a 1.4-fold increased risk of coronary heart disease (Steptoe and Kivimäki 2012). Workplace stress is also predictive of metabolic syndrome (Chandola et al. 2006) and major depressive disorder (Netterstrøm et al. 2008), and is associated with overeating, smoking, alcohol misuse, low levels of activity, poor sleep quality and social isolation (Chandola et al. 2008; Steptoe and Kivimäki 2012). The reduction of work-related stress is important for tackling stress-related health risks (Milczarek et al. 2009). Interest in the effectiveness of mindfulness-based approaches in the workplace has been growing given their potential to reduce current stress and protect against the effects of future stress (Wolever et al. 2012).

Mindfulness can be defined as a form of metacognitive monitoring of present moment experience without fixation or judgement (Kabat-Zinn 2009; Lutz et al. 2008). One’s ability to be mindful can be improved through training, and usually via an eight-week, structured group programme in which formal meditation practices are taught to foster accepting awareness of thoughts, emotions and body sensations. Sustained rehearsal of these practices appears to engender a disposition to be mindful in daily living (Chambers et al. 2009). Mindfulness training produces significantly different cardiovascular and autonomic effects than relaxation training (Ditto et al. 2006; Jain et al. 2007) and is thought to generate positive effects through distinct psychological mechanisms. The core proposed change is in the nature and function of attention (Bishop et al. 2004; Carmody 2009; Lutz et al. 2008), particularly the directing of attention and the monitoring of distracting thoughts, emotions or sensations (Jha et al. 2007; van den Hurk et al. 2010). Improved attentional control, when combined with awareness (Reb et al. 2013), is thought to be the building block for other changes pertinent to stress reduction including enhanced body awareness, emotion regulation, tolerance of negative states and de-centering (i.e. perceiving the self as an observer rather than casualty of stress experiences) (Carmody and Baer 2008; Hölzel et al. 2011). When sustained, these changes are collectively conceived of as enhanced mindfulness.

Meta-analyses of the effectiveness of mindfulness interventions on mental health and psychological distress in non-clinical populations report post-treatment summary effect sizes in the medium to large range (Chiesa and Serretti 2009; de Vibe et al. 2012; Grossman et al. 2004; Khoury et al. 2015). Variants of mindfulness interventions have been developed for implementation in organisations (e.g. Good et al. 2015; Klatt et al. 2009), and their effectiveness in reducing stress been indicated (Allen et al. 2015; Hyland et al. 2015) among working adults exposed to high occupational stress, including doctors, nurses and other healthcare professionals (e.g. Bazarko et al. 2013; Irving et al. 2009; Martín-Asuero and García-Banda 2010); teachers (Emerson et al. 2017); those working in occupations with high emotional labour (Hülsheger et al. 2013); and with indices of poor mental health (Huang et al. 2015).

However, whilst several studies have reported associations between increased dispositional mindfulness and positive outcomes (e.g. Baer et al. 2012), others have shown that not all currently measurable facets of mindfulness explain the effects of interventions on well-being (e.g. Eberth and Sedlmeier 2012; Nyklicek and Kuijpers 2008). We have little understanding of what these others factors are, and for workplace interventions in particular, as most studies have focused on outcomes rather than process. Where studies of mechanisms of change exist, they have tended to focus on clinical populations, where the application of mindfulness training (e.g. to coping with pain or cancer) is likely to shape process and outcomes (e.g. Dobkin 2008; Mackenzie et al. 2007; Malpass et al. 2012). Only a handful of qualitative studies have explored the experience of mindfulness interventions for non-clinical populations, and these have relied on feedback forms (e.g. Morone et al. 2012) or have reported experiential themes rather than mechanisms (e.g. Beckman et al. 2012; Cohen-Katz et al. 2005). Mechanisms of change have been explored for healthcare professionals but these have focused on the ways mindfulness can promote patient care or prevent compassion fatigue (Irving et al. 2014; Morgan et al. 2015). Experiences of change in a non-clinical, non-healthcare workforce have not been examined; many have argued that examining such experiences could offer theoretical developments about how mindfulness-based interventions are working within a normative, stressed workforce and how the nature and form of such interventions could be enhanced (Good et al. 2015; Hyland et al. 2015; Jamieson and Tuckey 2016).

The present study thus elicited retrospective, experiential accounts from people who had taken part in a workplace mindfulness-based intervention in order to generate a data-driven, provisional model of how positive benefits were secured by them. We examined an adapted mindfulness programme available for free to the workforce of a large higher education institute (HEI) in the United Kingdom. In the UK, academic and academic-related staff have reported high levels of psychological stress (Kinman et al. 2006), reports matched by other national and international studies of HEI workforces (e.g. Tytherleigh et al. 2005; Watts and Robertson 2011).

## Method

### Participants

Ethical approval for this study was awarded by a university research ethics committee (reference 12-0121). Participants were graduates of a workplace mindfulness-based intervention, described below. Every graduate from courses delivered between 2011 and 2012 (*n* = 59) were invited to take part in an interview with the following study framing: “We are interested in your experience of the course and the ways in which it may have influenced your self-awareness and ways of managing stress. Although the research evidence for the effectiveness of mindfulness is considerable, there is still much to learn about the factors influencing outcomes and how people apply it in their everyday working lives. We would like to learn about your experiences in order to make recommendations for the ways in which the University might develop mindfulness-based interventions for staff to improve personal effectiveness and reduce the negative impact of stress.” In total, 21 people consented to participate (*n* = 15 female; *n* = 6 male; *M* age = 47.0 years; range 26–61 years). Interviewee occupations spanned academic/research roles (*n* = 10, 47.6%), professional service roles such as in management and finance (*n* = 8, 38.1%) and clerical/ student support roles (*n* = 3, 14.3%). The length of time since intervention completion and the interviews ranged from 6 to 16 months (*M* = 10.9 months). Nine participants attended all 8 sessions (42.9%), 8 attended 7 sessions (38.1%) and 4 attended 6 sessions (19%). Sixteen of these (76.2%) had signed up for maintenance/booster sessions; these were two-hour sessions, once per month, which offered guided practice and group inquiry, similar to that on the intervention.

### Procedure

Since its inception in 2011, the intervention, called “MBSR-Mindfulness at Work”, had been offered by the Staff Counselling and Psychological Support Service (SCPSS) of the HEI as a stress reduction and well-being programme for all employees. Provision of the programme reflected an organisational intention to deliver more illness prevention, early intervention and well-being support for free to the workforce. The intervention was delivered solely by SR (second author) who had completed Mindfulness Teacher Development Training (Centre for Mindfulness Research and Practice, Bangor University). SR adhered to the Good Practice Guidelines for Teaching Mindfulness-Based Courses developed by the UK Network for Mindfulness-Based Teachers (2010).

“MBSR-Mindfulness at Work” was a workplace adaptation of Mindfulness-Based Stress Reduction (MBSR) (Kabat-Zinn 1990, 2009). Delivered over eight weekly sessions, the intervention included the following: psycho-education (e.g. stress response, metacognition); guided meditations (e.g. body scan, breathing, movement and sitting meditations); and group inquiry. Participant resources included CDs of guided meditations and home practice logs. Adaptations from the standard programme were as follows: shorter sessions (2 rather than 2.5 hours); exclusion of the full practice day; and inclusion, in sessions 4 and 5, of the workable ranges model of stress regulation (Rose 2014; Rose et al. 2017) which is used by the SCPSS as a psychoeducational tool. The model proposes a “window of tolerance” (Siegal 2010) and optimal functioning between two different reactions to stress, namely highly charged mobilisation and low energy immobilisation.

Semi-structured, audio-recorded interviews were conducted by the fourth author on university premises. The interview explored the following: participants’ interest, motivations and expectations pre-intervention; their conceptualisations and experiences of workplace stress; their experience and learning on the intervention; key moments/turning points on the intervention; their achieved understanding of mindfulness; influences on work, stress, and physical and mental well-being; the long-term sustainability of outcomes; ongoing support needs; and if the availability of the intervention influenced their perceptions of their employer. Interviews ranged from 57 to 101 minutes (*M* = 65 minutes; SD = 14.56). Audio recordings were transcribed and anonymised prior to analysis.

### Data Analyses

Analysis was an abbreviated form of grounded theory (Willig 2008), drawing upon methods described by Charmaz (2006) and Corbin and Strauss (2008). As the aim was a provisional and not exhaustive model of change, we did not utilise theoretical sampling or saturation. Grounded theory helps the researcher to move from a description of what is happening to an understanding of the process by which it is happening, meeting the study aim of generating a substantive (rather than formal) theory that makes sense of the data in its own context. The analysis was driven by the following questions: what was the experience of participating in the intervention?; how was any change or impact experienced, and what was its nature?; were there experiences which appeared to be prerequisites to further change? We analysed those aspects of the narratives that pertained to the intervention experiences (rather than post-intervention) that appeared to explain how positive outcomes were secured.

Analysis involved a first stage of open coding, which involved line-by-line labelling of text segments, from which provisional descriptive categories (e.g. the effect of the group) or, where data permitted, interpretative categories (e.g. normalisation of the stress response) were generated. Provisional categories were then compared, modified and/or renamed as more data was coded. Axial coding was the second stage of analysis, and involved exploring category connections across the data set, being attentive to what may be underpinning their connection and what phenomena (labelled as conceptual themes) might be central to the experience (e.g. that the intervention facilitated increasing awareness of opportunities to respond differently). The final analytic stage was theoretical development, which positioned conceptual themes in relation to each other in a way that fitted participants’ accounts as closely as possible (e.g. that accepting the importance of self-care was an early foundation for positive outcomes). Whilst grounded theory aspires to be atheoretical, in practice, analysts are usually sensitised to dominant concepts in the field. We managed this by scrutinising interviewees’ use of apparently familiar terms (such as attention and awareness) for their nuanced nature and purpose in their accounts, and if and how they differed from the dominant use of the terms in theory and practice.

As the first and third authors had previously attended the Mindfulness at Work intervention, and the second author designed and delivered the intervention, an independent researcher (the fourth author), who had not attended the intervention, was appointed to conduct the interviews and complete initial coding. The emergent analysis was then discussed with the other authors collectively, and in progressive stages, in order to refine emergent conceptual themes and their position in the final model. As is commonplace in qualitative work, the researchers attempted to be cognisant of their unique orientation to the data, particularly given their differing levels of engagement with the intervention. Group discussion was an important mechanism by which we explored and, where necessary, neutralised any detected bias whilst also attempting to capitalise on the “insider-view” available to three of the researchers. For example, the first author was noted to be particularly drawn to internal experiences of de-centering and was somewhat less alert to participants’ accounts of how these were deployed in the workplace; the third author (an organisational psychologist) was able to ensure we remained attentive to how these experiences worked in practice for participants. Developments in categories and themes were systematically logged and audit trails produced from the first-stage coding to the final model.

## Results

**Fig. 1:**
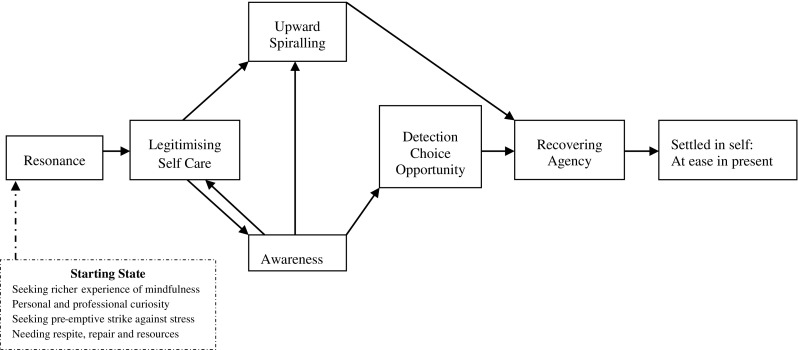
Provisional model of change

Although many grounded theory outputs propose a core category, the present data did not point to this. Rather, participants’ accounts suggested a staged process with identifiable starting and end (or at least current) states with shifts occurring when one stage gave rise to new ways of thinking and/or experiencing which were felt to be distinct from what had gone before. We thus offer a broadly temporal, linear model (see Fig. 1) with some interdependent relationships. The model proposes that the arising of new experiences builds on previously gained insight and experiences. Although early intervention experiences are likely to remain important throughout the intervention, we present them as discrete, initial stages of change since they appeared to be important building blocks for subsequent benefits. We describe each stage of the model with illustrative interview extracts from across the study sample. Table 1 indicates which participants contributed to each category.

**Table 1:** Individual participant contribution to model components

	Reported motivation for programme attendance				Participant contribution to components in the model of change						
Participant	Develop a richer understanding (*n* = 13)	Personal/professional curiosity (*n* = 14)	Pre-emptive strike (*n* = 17)	Seeking respite, repair and recourses (*n* = 13)	Resonance (*n* = 9)	Legitimising self-care (*n* = 10)	Awareness (*n* = 18)	Detection-choice-opportunity (*n* = 13)	Upward spiralling (*n* = 10)	Recovering agency (*n* = 20)	Settled in self: at ease in present (*n* = 10)
1	✓	✓	✓	✓			✓	✓	✓	✓	✓
2		✓	✓	✓	✓		✓	✓		✓	
3		✓	✓		✓	✓		✓	✓	✓	
4	✓	✓	✓	✓	✓	✓	✓	✓		✓	✓
5		✓			✓	✓			✓	✓	
6	✓	✓	✓				✓		✓	✓	
7	✓	✓	✓			✓	✓			✓	✓
8	✓						✓			✓	
9	✓	✓	✓	✓		✓	✓	✓		✓	✓
10	✓		✓				✓	✓	✓	✓	
11	✓		✓	✓			✓	✓	✓	✓	
12	✓			✓	✓	✓	✓		✓	✓	✓
13	✓		✓	✓		✓		✓		✓	✓
14		✓	✓	✓		✓	✓		✓	✓	
15	✓	✓	✓	✓	✓		✓	✓	✓	✓	✓
16	✓		✓	✓	✓	✓	✓				
17		✓	✓	✓			✓	✓		✓	
18		✓	✓	✓	✓		✓			✓	
19		✓					✓	✓	✓	✓	✓
20		✓	✓	✓	✓	✓	✓	✓		✓	✓
21	✓		✓				✓	✓		✓	✓

### Starting State

Reasons for attending the intervention included: wanting to develop a richer understanding of mindfulness; personal and professional curiosity; wanting to make a pre-emptive strike against stress; and a need for respite and repair from stress. Taking a pre-emptive strike against stress was talked of in terms of improving one’s affective and cognitive regulation in a context of increasing organisational demand: “even though most of the time you’re quite resilient to it” (9), one can get caught up in problems with “concentration, perfectionism, procrastination [...] and you get to the point where you can’t do anything effectively” (2). Seeking proactive stress management indicated that personal resources were finite and needed to be replenished: “I was aware that my stress levels were rising and I didn’t want them to [...] you can never know too much about how to take care of yourself” (11). Thirteen participants were seeking different solutions to their stress experiences, namely; (i) repair: “I just needed to invest in myself so I could keep me, me and work,work.” (9); (ii) respite: “I think more than anything it was time, time out from everything that was happening” (15); and (iii) resources: “anything that looked at reducing anxiety and stress levels that didn’t involve medication” (1).

#### Stage 1: Resonance

The proposed first stage of change is an experience of resonance. This could be in terms of *intellectual* resonance, whereby the psycho-educational components of the programme makes sense to people (“I need the evidence and I liked that and I kind of engaged with that more because I kind of understood how it worked” (19); “it all clicked with me quite well, right from the beginning […] it fitted into a framework that made sense” (4)) or *experiential* resonance, whereby participants have an early embodied, experience of mindfulness: “after the session, and after some practice, it just felt like this ton of whatever it was on my chest, yes, heavy weight of anxiety was lifted it was just so so liberating”(16); “I was a bit curious about why we needed eight two-hour sessions of the course to sit there. And then two sessions in, I realised why” (2). Another participant recalled a new experience, in the first session, which drew her in: “the exercise made the raisin taste so phenomenal […] that’s really stayed with me from the first session” (15). Resonance was also supported through group validation as “everybody was experiencing some kind of stress, and there was that shared experience” (15). Hearing others’ accounts of workplace stress had an engaging and normalising function: “it does give you a sense of comfort to know that what you’re experiencing is not uncommon” (15). This resonance facilitated “a sense of connectedness” (5) where it was “nice to look around and there are people there that get why it [mindfulness] is good” (12), enabling participants to “talk about it quite a lot […] what we were getting out of it” (19). For many, this resonance was felt to support the emergence of acceptance and compassion at a group level: “the shared experience which was quite nice, because I felt kind of quite isolated at the time (15)”, “it was very reassuring; it was a very comforting place” (6).

#### Stage 2: Legitimising Self-Care

Legitimising self-care is the proposed second stage of change. For some, attending the programme reflected a decision to invest in their own well-being. For others, a ‘realisation’ of the importance and benefits of self-care came via the early sessions of the programme, and programme attendance became, for many, a demonstration of self-care (e.g. “I’m allowing myself that hour of paying attention to me completely”, (4)ˑ“I’m sort of valuing myself enough to say this is important” (5). Nonetheless, there appeared, for many participants, to be a process of intellectual justification, whereby taking care of one’s mental and physical health needed a logical defence. This justification seemed greatly supported by the fact that the programme was embedded in, and provided free by, the organisation: “I think it legitimised self-investment. […] if you don’t look after yourself, you’re not well […] In this organisation, in this University, it legitimised self-management and looking after yourself and being nice to yourself, as opposed to always being nice to other people, so that helped me then invest some time” (9). Another participant stated: “the fact that the University ran the course seemed to be saying it’s okay to take care of yourself; it gave you the right to do it” (9). It was, for one participant, an indicator of “a quiet revolution” (16) in terms of organisational investment in mental health care, with others reporting increasing affiliation with the organisation: “we sort of felt connected to the University” (5): “it’s not so common to see that your own employer actually cares about the way you feel, cares about you as an individual” (3). Acknowledging the need for positive action for personal well-being appeared to be an important facilitator of personal mindfulness practice: “To ‘look after me’- the idea that I have to do it and not somebody else and that there is time in my busy schedule” (20); “even seeing it [practice prompt] in my diary would be kind of a reminder, I suppose in itself, that this is a practice that I’m trying to use to help me” (14). Thus, although legitimising self-care was an ongoing process (e.g. “there is time in my busy schedule”), it is presented here as an early first stage of change as it appeared foundational to intervention engagement and the securing of subsequent benefits.

#### Stage 3: Awareness

Developing a new awareness of the state of one’s mind and body, as well as one’s habitual ways of responding, was expressed as a fundamental mechanism by which mindfulness practice opened up new choices for responding and new opportunities for ways of being. This new relationship between awareness, facilitating detection (of stress) then choice represents the third and fourth stages in the proposed model. During the intervention, participants reported previously having “inhibited and denied my physical and emotional responses to different situations” (15) or being “quite instinctive” (1), many now felt “more aware of my own thought processes and responses to things” (1): “I wasn’t making any changes but I was becoming much more aware” (15). Participants recounted new abilities to “track my focus, my concentration, my breathing…being really bodily aware and sort of seeing what was happening” (17) and to “centre on yourself to really know how you’re feeling and how your thoughts are” (21). Becoming more self-aware also shaped participants’ motivation to engage in self-care.

#### Stage 4: Detection-Choice-Opportunity

Increased awareness facilitated earlier than usual detection of psychological or physiological markers of stress: “I’m much much quicker in detecting that specific [stress] mindset and that physical [reaction] then stopping it by means of paying attention to the body” (10). Earlier detection of state changes meant that participants had a different potential for action, either cognitively, affectively or behaviourally: “I now have a chance to detect and be aware of how I am in different situations. Then you have different opportunities to go different ways at that point. If you’re not aware, you’ve no opportunity to choose what to do. That’s a very important connection” (20). Whilst some felt that their application of mindfulness principles in the workplace was **“**part of my day to day routine” (3), others reported that this was a new opportunity to respond to acute stress or panic differently: “the sooner you can catch your acceleration or you’re, you know, not feeling so good, then the sooner you can do something about it” (17); “it helped me to recognise that a lot of strong, difficult feelings are very short-term and actually by not focusing on it in my head […] but by focusing on what it feels like physically, really helped” (14). Others reported new opportunities to respond differently in the workplace in terms of affect driven behaviour: “I do remember on that occasion wanting to desperately respond immediately and going, no I don’t need to respond immediately, take some time” (15); “[meetings] can become very, very emotional and very, very argumentative [...] it (body scan) was an interesting exercise to apply in those meetings” (3)**.** Others talked of cognitive shifts: “just letting them [problems] fall into perspective” (2). Implicit in these accounts of new ways of behaving or experiencing were a range of self-regulation, re-perceiving, acceptance and decentring processes: “it’s being in the present moment, the centring, to actually think “this is now” [...] and it’s the acceptance of events” (21): learning “to not respond right away”(2); “accepting people for who they are” (19); “you can’t do anything about how people act [...] you need to be able to reflect quickly and not to take it personally as well which is also where I think this course was useful” (3). Thus, participants expressed both the motivation and ability to apply mindfulness across their working life to enable them to choose new responses.

#### Stage 5: Upward Spiralling

Once the core principles of mindfulness were accessible to participants, a process of upward spiralling emerged. This is represented as the fifth stage of change, influenced directly by self-care, awareness and choosing new ways of responding. It seemed that participants began to ‘test’ mindfulness and seek applications themselves in their day-to-day lives: “it’s a personal exercise as well, you need to put it into context into your own environment, you know, to really learn from it” (3). Experimenting in real-life gave rise, for some, to assimilation (e.g. equating mindfulness with existing healthful practices, such as exercise) or accommodation (e.g. completely reframing experience or understanding). These times of experimentation and learning not only reinforced the relevance of mindfulness, but opened up new ways to access calmer, more accepting, and sometimes more positive states of being: one participant reported experimenting with self-talk such as “it’s good enough” and “slow down” (6). Another reported walking across campus in the rain to a meeting, noticing their ruminative state and intentionally testing what would happen if they tuned into present moment awareness of sounds. This experiment with mindfulness “changed my mood a lot” (11) and shifted their thinking about the fixedness of experience. Another participant tried to use mindfulness to be more present during their holiday, when normally their mind would be “churning away” (1). Testing the way that one can “use my body to fix my mind” (6) was common, with participants having moments of insight whereby “although I knew that intellectually, it wasn’t until I tried it that I then thought, yes, this actually works” (6). These tests of mindfulness appeared encouraging (“I felt it working”: 3) and at times, profound: “I felt energised [...] with a sort of clarity of mind, and I remember feeling, if this is how I can be in my day-to-day work and home life, I’ll be a better person” (1).

Choosing to bring positive experience into high resolution, or “becoming more sensitive to all the good things out there” (6) was, for some, a further constituent of the upward spiralling process: “because I was paying attention I actually noticed this rather beautiful sound and got a nice experience for myself, where before I would have been caught up in all the negative thinking” (11); “Actually focusing on the here and now and enjoying that moment of being aware of it [...] raising your consciousness about that, which, there’s something very good about that” (6). Such present moment connection to positive experience led many participants to describe mindfulness as life-affirming: “there are all these different moments that we can have […] it’s about connecting with all those little pleasurable moments that we miss” (11).

Finally, continued, intentional self-care appeared to not only prevent relapse into negative states, but contributed directly to upward spiralling, whereby simply choosing to practice mindfulness was experienced as a positive, beneficial act: “you’re looking after yourself and you’re nourishing yourself and doing something positive” (12), as well as using increased self-awareness to be compassionate to oneself: “I was learning to be kinder to myself” (15). One participant felt this was a key learning experience for her: “if I am feeling a bit low I think right, what can I do that’ll make me feel, what’s a nourishing activity? [...] it feels nice to look after your mental health in the same way you would your physical health” (12).

#### Stage 6: Recovering Agency

With increasing confidence that mindfulness ‘works’, and with a more positive and hopeful state of being, participants seemed to experience a renewed ability to cope with work demands and stress. This appeared very important, given that many had expressed feeling diminished or without resources to deal with stress: “I learned to be more in control” (2); “I feel like I manage things more” (12); “you’re realising that’s not controlling you, you can control it” (14). This recovery of agency is represented as a sixth stage of change. Many connected this renewed sense of control to the feeling that they now had a toolkit for life: “like a coping strategy, it [mindfulness] is a tool that I’ve got, I know I can switch off […] its giving my brain a break […] it’s how I can deal with my life actually (12): “I now have some habits, or useful tools” (14); “I was learning useful methods for keeping the bad thoughts at bay” (4). The particular ‘tool’ that seemed to help participants most in this regard was the formation of a new relationship to thoughts, particularly useful when participants were experiencing negative states: “I learnt that the bad thoughts did not reflect reality, I learnt that they were mental events [..] I’d put on the headphones and do one of the guided meditations and that would be effective at keeping the bad thoughts off” (4). A new tendency to observe or be aware of one’s mental state was helpful to some, enabling them “to see what the internal chatter was all about” and when their mind had “attached itself to whatever”(17), knowing they had strategies to circuit break unhelpful thinking, usually by decoupling from emotional and cognitive states: “remembering that actually this isn’t my stuff…if I react to it, then it is my stuff” (14); “I consciously let it go” (10). Thus, using mindfulness principles along with heightened awareness evoked important changes in their sense of agency.

Mindfulness as a toolkit for agency was also talked about in terms of improved clarity of mind, helping participants to feel they were able to get themselves “back on track” (12), and out of either stagnant or overwhelming work experiences. Many described feeling more in control of their minds at work through mindfulness practise: “it retrained me to listen […] I became more thoughtful at work” (1); “I didn't feel I was struggling the way I did before. I was kind of unclogging everything emotionally - it had a knock on effect on my work as I’m working effectively and getting loads done, and it doesn't feel overwhelming”(12). Reported benefits included improved time management, planning, clarity and productivity: “asking myself what do I want to accomplish today? To clear the mind and stop fiddling” (2); “[a mindful practice] is like a nice detox, it just settles your mind, and then I’m alright and I’ll carry on” (9); “it was improving my working practice, really, which is what I hoped to get out of it” (1). Using mindfulness practices in response to troubled sleep was reported as effective, and meant, for some, an improved ability to deal with the next day. These experiences helped participants to feel back in control, with now personally evidenced-based resources to help them regain more balanced states of mind, clarity of thought and more helpful responses to others.

#### Stage 7: Settled in Self: at Ease in the Present

Participants reported changes over time that were fostered by learning, group interaction, self-reflection and experiences of what happens when one attempts to be mindful. For many, transformational change was reported: “it’s completely changed my life” (12); “it’s been life changing” (21). The ‘end state’ of participants at the point of interview differed in many ways (e.g. full commitment to mindfulness, ongoing need for support, using mindfulness minimally) although the reasons for this (beyond individual differences) are unknown. However, there were also commonalties among participants in their ‘end state’, and these are reflected in the final stage of the model termed *settled in self: at ease in the present*. *Settled in self* represents finding a new way of relating to the self which felt at ease (“it’s just sort of given me confidence to be comfortable with my own brain [...] it seemed to be something that wasn’t difficult, that we all must have within us”:1) and was often equated to “coming home”:“it’s almost like finding yourself” (21); “I don’t know if its spiritual […] I can’t quite explain what it is but it’s something that I almost like home into, I’m homing into that” (16). *At ease in the present* represents a new way of relating to everyday events with acceptance: “it really is being able to accept that things aren’t always great” (19); “It’s that acceptance of life events […] it gives you strength to cope with whatever comes your way” (21).

Thus, our provisional model proposes that workplace mindfulness interventions may reduce workplace stress and engender well-being though subtle, interactive, and progressive stages of change. Although the model maps a temporal course of change, the experience of change was likely integrative. As participant 2 stated: “you get towards the middle [of the intervention] and you realise you’re not just accepting things, you’re kind of moving things about so that you can see them differently” (2). The model also does not incorporate challenges or barriers to engaging in, and practicing, mindfulness. These were not systematically interrogated in the interviews, but some participants reported: the challenges of finding time to engage in meditative practices at home and at work; that the lack of private space at work prohibited practice also; that taking time to complete a mindfulness practice often led to feelings of guilt about what they could have achieved in that time; that being highly stressed depletes capacity to engage in mindfulness practice: “once it takes hold, it inhibits my capacity to draw on any strategies. My cognitive space is occupied with thinking through problems, preparing scenarios, anticipatory anxiety” (7); that changing engrained ways of thinking and behaving is hard; that more personal evidence of effectiveness in the workplace was needed; and that applying their learning to real life can be challenging (due to the inability to concentrate).

## Discussion

Based on participants’ accounts of a workplace mindfulness-based intervention, this study proposes a provisional model of change to explain how the intervention appeared to help participants secure meaningful benefits. Our proposed model is consistent in several ways with other accounts of how MBSR secures positive outcomes and it partly aligns with the MBSR curriculum in terms of first developing attention and awareness. However, as far as we are aware, this study is the first to report that early *resonance* (between the programme and the person) may be an even more foundational mechanism of change, at least in workplace mindfulness programmes. Many of our participants wanted the intervention to be justifiable intellectually and from the outset. Psychoeducation components and the group were important in meeting this need, typically by representing both the psychophysiology and social views of stress in ways that made sense to them. Other participants reported a less analytic, but equally persuasive experience of resonance in the very first session, described as feeling mindfulness ‘working’. These different representations of resonance may be reflective of dual information processing models of the self, which posit that reality can be processed via an analytical-rational system and/or an intuitive-experiential system (Pashko 2016). The former is slow but conscious and the latter is quick but unconscious and pre-linguistic, and it has been proposed that people vary in their preference for each (Epstein et al. 1996; also described as a “need for cognition”; Norris and Epstein 2011). Our data suggested that intervention participants may come with differing processing preferences and that the ability of MBSR-based interventions to meet such individual differences in the first session may be important to securing subsequent benefits.

The second stage of *legitimising self-care* captured participants’ need to justify active care of their mental health. Although *legitimising self-care* is presented in the model as an early experience foundational to later benefits, it was an ongoing process for many participants. We present it as a discrete early stage as it was an early experience reported by most participants and appeared to be a necessary building block for subsequent experiences. A sense of reluctance and/or guilt for engaging in self-care has been reported in many mindfulness studies in diverse contexts and countries (Beckman et al. 2012; Irving et al. 2014; Morgan et al. 2015). The modelling of self-care in the group appeared to help our study participants. Group effects in MBSR-based interventions are expected, and reported, as vicarious learning, normalisation, cohesion, empathy, compassion and reduced professional isolation, and are therapeutic in stress reduction in and of themselves (e.g. Beckman et al. 2012; Dobkin 2008; MacKenzie et al. 2007). Irving et al.’s (2014) model of change, based on healthcare practitioners, similarly positioned group experiences as an intervening factor, whereby peer endorsement of the intervention’s application to working life improved the intervention’s credibility and acceptance. Notably, our participants also reported that it was the organisation’s provision of free and easy access to the intervention, and thereby its representation of support for staff well-being, which also strongly legitimised their participation. Thus, the way the intervention came to have positive effects appeared, at a foundational level, to be bound up with its implementation in the workplace. There is some evidence that, compared to out of hours delivery (e.g. van Berkel et al. 2014), providing mindfulness for stress reduction in the workplace during working hours may be particularly beneficial for a stressed workforce (Duchemin et al. 2015; Horner et al. 2014; Huang et al. 2015). Thus, given the proposed two first stages of change, the present study’s findings pose new questions about how best to support the resonance of mindfulness- based interventions with members of a general workforce who may benefit from such programmes but who may be hard-to-reach because of scepticism about mindfulness and/or the benefit of self-care and preventative action.

In our provisional model, *legitimising self-care* directly fosters *awareness.* This is different to Irving et al.’s (2014) model whereby self-care was positioned as an outcome rather than also as an intervening stage. Additionally, although attention is typically dominant in mindfulness models of change, participants in our study talked primarily about awareness (of self) rather than attention; thus, attention does not appear explicitly in our model. However, attention and awareness are interdependent, with attention driving an experience of awareness, and awareness informing attention (or lack of it), and it is possible that awareness was simply a more usable construct for participants, as also noted in Irving et al.’s study (2014). Improved self-awareness is well established as an outcome of, and mediator of change in, mindfulness programmes (e.g. Morone et al. 2012). Participants’ accounts in this study resonate with existing findings that now being able to “see what was happening” in their bodies, thoughts and emotions facilitated change, suggesting that their baseline levels of self-awareness were low. The fourth stage of change, *Detection-Choice-Opportunity*, represents the ways in which this increased awareness of, and ability to detect changes in stress physiology (also termed ‘body awareness’; Hölzel et al. 2011) and emotional or cognitive states at work, provides an opportunity for people to alter their relationship with those states, and subsequently makes available the prospect of engaging in alternative cognitive, affective or behavioural responses. Thus, awareness of inner experiences appeared to be an essential precursor to further change and benefits.

The intention to increase self-awareness in order to reduce stress is not unique to mindfulness interventions. Psychodynamic approaches, for example, cultivate an “observing self” or meta-awareness (Schooler et al. 2011) to defuse automatic thinking or feeling in order to interrupt the stress response (Williams 2010). What may be unique to mindfulness training is the practice of a particular attitudinal response to awareness, as reported by our participants. Practising new ways of relating to conscious experience is fundamental in mindfulness training and can include attitudinal change (e.g. curiosity, kindness and acceptance; Keng et al. 2012), as well as cognitive re-orientation (e.g. distancing, de-centering and re-perceiving; Shapiro et al. 2006). These new ways of encountering experience are often taken up and interpreted by participants as new “coping strategies” (Irving et al. 2014; Morone et al. 2012). As argued elsewhere, when awareness is coupled with these kinds of strategies, de-automatisation is more likely (Kang et al. 2013; Reb et al. 2013). This refers to the minimisation of automatic, largely unconscious processes (driven by habit or heuristics) to inform the interpretation of experience. This is represented in our model in terms of *Detection-Choice-Opportunity*, as this was the way in which participants operationalised the construct. Consistent with other qualitative studies, our findings show that mindfulness training and application to daily work life, weakens the automaticity of and engagement in habitual, negative ways of experiencing and responding to stress, increases people’s resources to cope and supports a faster return to baseline (Cohen-Katz et al. 2005; Irving et al. 2014; Morone et al. 2008, 2012).

*Upward spiralling* is the fifth proposed stage of change, supported by participants’ experimentation with “being mindful” in real world situations. Our provisional model proposes that these positive gain cycles are influenced by *awareness* and *self-care*. In addition, the intervention’s encouragement, through the pleasant experiences exercise and more generally, to “bring positive experience into higher resolution” appeared to help in that, by broadening people’s attention to good moments, stress burden decreased. Such ways of altering day-to-day appraisals appear similar to the notion of benefit-finding (e.g. Garland et al. 2011), itself associated with changes in stress physiology (Bower et al. 2008). Upward spiralling is represented in other models of mindfulness wherein mindful coping is proposed to facilitate positive psychological processes that build resilience (e.g. Fredrickson and Losada 2005; Garland et al. 2011). Although tentative, their work suggested that mindfulness operates by strengthening positive cognitive-emotional processes rather than by disrupting negative ones (e.g. catastrophizing). Our model only partly concurs with this proposition as *awareness* and *self-care* appear to both inculcate positive, and disrupt negative, cognitive-emotional processes. For example, participants reported dynamic processes whereby, as awareness became more routine, so did self-care. They reported being more able to interrupt a stress experience and replace it with a beneficial activity (such as break-taking or exercise), pointing to a possible mechanism by which mindfulness training may come to build resilience to stress-related mental health difficulties. Other evidence suggests that transitory states of mindfulness, when repeatedly induced every day, may engender trait or dispositional mindfulness (Chambers et al. 2009; Garland et al. 2010), which would manifest as a new way of coping with stress. Of course, that many participants attended the intervention for stress reduction is likely to have influenced their attention to the disruption of negative cognitive-emotional processes.

*Recovering agency* refers to participants’ accounts of increased control compared to their pre-intervention state of feeling overwhelmed and without coping resources. Clinical studies often report increased self-control as an outcome of mindfulness training (Dobkin 2008; MacKenzie et al. 2007), and it is implicated in Irving et al.’s (2014) model as part of strategies and consequences. Recovering agency was talked about by participants more than any other stage of change, and it was expressed as evolving over time, supported by *detection-choice-opportunity* and *upward spiralling.* Many participants talked of now having “toolkits” (a common description following MBSR, e.g. Morone et al. 2012) for improving working practices and managing stress triggers and stressful episodes. Their accounts reflect key features of the Conservation of Resources (COR) theory whereby psychological health is theorised to require a strong armamentarium of social and personal resources, the input-output balance of which must be vigilantly managed (Halbesleben et al. 2014). Our study showed that knowing one has a mindful “toolkit” is perceived as a psychological asset (e.g. see Youssef and Luthans 2007). Although some studies failed to find an effect of mindfulness interventions on job control (when assessed via short standardised measures; e.g. Huang et al. 2015), in interview, our participants reported feeling new equipped and able to apply mindful practices and principles to challenges in working life.

At the time of interview, most participants reported that mindfulness training had engendered a sense of being s*ettled in the self: at ease in the present*. That mindfulness brings about a sense of peace, calm, well-being and even serenity is well documented (e.g. Liu et al. 2015; Morone et al. 2012), yet little empirical work exists on the mechanisms by which mindfulness practice fosters this outcome in particular. Our findings suggest that a sense of peace was influenced by being settled in who they are, and by practising acceptance of the way things are. Acceptance of self and experience is an intended outcome of mindfulness training, and seems important to a sense of well-being, a proposition supported by Xu et al.’s (2015) study in which the positive association between mindfulness and peace of mind was mediated by self-acceptance. It has been argued that equanimity (a concept related to acceptance), defined as an even-minded mental state toward all experiences, internal and external and regardless of their valence (Desbordes et al. 2014) can, over time, become an effective counter to allostatic load following chronic stress (Karatsoreos and McEwen 2011).

Overall, our provisional model proposes stages of changes that may be experienced by people on an adapted workplace mindfulness-based intervention for stress reduction. The proposed stages of change point to experiences not routinely measured in mindfulness interventions, including the importance of early engagement and resonance, intentions for self-care, recovery of agency and a sense of peace. Notably, our findings support Good et al.’s (2015) hypotheses of how mindfulness might promote well-being at work, including the de-automatization of potentially toxic responses (seen in *detection-choice-opportunity*), increased confidence in dealing with challenging workplace situations (seen in *recovering agency*) and greater experience of positive emotions (seen in *upward spiralling*). In addition, whilst the present study cannot constitute an analysis of mindful emotion regulation, our data point to the possible unique contributions of group- and workplace MBIs here. Mindful emotion regulation has typically been conceived of as a principally internal process (Chambers et al. 2009; Hölzel et al. 2011). However, our findings suggest relational and organisational influences on early stage emotion regulation processes. Specifically, psychoeducation and the effect of the group appeared to facilitate intellectual resonance and normalisation of the stress response, which in turn helped people to re-frame their stress experience as modifiable. In addition, the personal and organisational legitimisation of self-care for stress management appeared foundational for investment in mindful practice. Together, these features appeared to help establish openness to new, mindful ways of relating to stressful emotions and thoughts. Such ways of thinking appear different to the intrasubjective emotion regulation process of re-appraisal often used in connection with mindfulness (e.g. Hölzel et al. 2011). However, participants’ foregrounding of the role of awareness and detection does support the central role of attention in emotion regulation established elsewhere (Good et al. 2015; Reb et al. 2013), and staying present with strong feelings and replacing reacting with intentional responding may correspond to exposure and extinction as described by Hölzel et al. (2011). Furthermore, upward spiralling, the recovery of self-agency and increased settled sense of self may relate to reconsolidation (Hölzel et al. 2011).

The proposed model may direct further examination of possible mechanisms of change. For example: how can early resonance be promoted to increase the intervention’s reach to those who are at risk of stress-related illness but unlikely to self-refer?; what can be done at an organisational level to foster sustained legitimisation of self-care for the prevention of stress-related outcomes?; and does the self-care endorsed in mindfulness-based interventions engender the uptake of other health promoting behaviours? Additional questions remain about the preventative power of mindfulness training. Although we know of participants’ frustrations and challenges in mindfulness training (e.g. Moss et al. 2008), future research should aim to identify why people might find it difficult to apply mindfulness training in the workplace, and whether ongoing mindfulness meditation is pivotal to sustained changes in stress experience. In addition, as our data prohibited possible explanations for the differing end state of participants, future studies could use our model to examine possible mediators and moderators of this, for example, whether the likelihood of establishing a consistent mindfulness practice is predicted by strong resonance during the intervention.

### Limitations

We evaluated the study against Tracy’s (2010) eight criteria for excellence in qualitative research and rated it high in terms of being (i) a worthy topic, (ii) credible, (iii) having resonance, (iv) a significant contribution, (v) coherent and (vi) ethical; and moderately well on (vi) richness and rigour (given the moderate sample size). As contemporary theory of knowledge disputes the possibility of a neutral observer, Tracey’s final criterion (viii), reflexivity and transparency, invites researchers to consider their influence on data generation and analysis, appreciating that we can only ever be partially conscious of this (Malterud 2001). The first three authors are likely to have been sensitised to the data in particular ways as SR designed and delivered the intervention and SHJ and GK were participants on the pilot delivery of the intervention. Whilst this familiarity with the intervention helped in contextualising participants’ accounts, it is possible that our view of mindfulness as beneficial, and as involving changes over time, meant we were likely to be particularly alert to similar experiences reported by participants. Given this closeness to the data, we assigned a fourth independent researcher (RSE) who was without connection to the intervention, to conduct the interviews and first-stage data analysis (i.e. coding to categories). Additionally, we sought to manage our influence on data analysis through constant grounding in the data and collective group discussions of emerging analytic thoughts. Barry et al. (1999) have argued that a team can improve the rigour of qualitative analysis and foster conceptual thinking compared to individuals working alone; our experiences resonated with this in that having four people involved in analysis precluded one dominant orientation to the data and prompted a good level of checking with the data to enable consensus.

A number of limitations to this study should be noted. Although there are multiple perspectives on the validity of retrospective reports (Schwarz and Sudman 2012) and the ontological positions available in relation to them (King and Horrocks 2010), accounts of the past can generate useful insights into the nature and meaning of experience for people. The retrospective narratives produced via interviews in the present study are likely to have involved recollection, reconstruction and co-construction. Thus, the proposed model of change is a highly subjective and probably incomplete one—although we were nonetheless able to identify patterns across participants. Furthermore, participants varied in the time since programme completion and interview participation; whilst we drew only on aspects of the interview data which clearly pertained to experiences on the programme (and often checked this was what participants were referring to), it is possible that participants infused their recollection of the programme with practices and benefits they had in fact secured post-programme. In addition, participants had opted into both the programme and to the interview study, and it is likely participants were positively biased. Thus, our proposed model represents a framework of change for a specific, motivated population for whom mindfulness was felt to “work” over a period of time. The model should be examined in diverse workforces to test its validity as other workforces, working under different conditions, stressors and resources may experience different mechanisms of change. As one of our participants reported, a HEI environment is “hypercritical, like that sort of critical ability is so integral to research and to teaching, and you, I mean I then apply that to every aspect of my life and, and myself” (15). Thus, workplace mindfulness programmes might work differently depending on the dominant workplace climates and concerns. In addition, future studies could use our model to examine possible mediators and moderators of end states, for example, whether the likelihood of establishing a consistent mindfulness practice is predicted by strong resonance during the intervention. Finally, whilst this study involved participants who had completed the intervention up to 16 months previously, there remains a need for substantially longer follow-up periods to determine if individual level changes are sufficiently robust on return to the workplace context (which remains unchanged) and its indigenous stressors.
